# 

**Published:** 1869-01

**Authors:** 


### INDEX TO VOL. X.

PAGE.
Address, Annual to the Montgomery Medical Society, by P. B. Cook, M.D., ---------------- 65
Ammonia Muriate in Syphilis, by T. Griffin, M.D., -------------- 13
American Medical Association, 123, 179, 182, 239, 246, 248, 342, 375
Alumni of Chicago Medical College, ------------178, 239, 257, 298
Aphonia, Rebelious cure of,----- 181
Academy of Sciences of Paris,--- 183
Amputation of Arm in Utero, by Mental Emotion,------------- 561
Address, Introductory, by H. W. Boyd, M.D.,---------------- 641
Ammonia Muriate, in Ovarian Neuralgia, ----------------- 651
Animal Quinoidin, Doctrine of,-- 170
Auscultation and Percussion, Compendium of,----------------- 237
Archives de Physiologie, Normale et Pathologique,-----------238, 634
Arsenic in Diseases of the Skin,-- 765
Antiseptic Surgery, Sum and Substance of,-------------------- 732
Aneurism, Treatment of, -------- 751
Ages of the Ancients,----------- 762

Blood, Transfusion of—Succcessful Cure,---------------------183, 766
Best Book for Everybody,------- 184
Blood-letting, Action of, by Geo. Johnson, M.D., -------------- 234
Bromide Potassa for Sleeplessness of Infants, ------------------ 251
Bromides, an Inaugural Thesis,-- 263
Bromide of Ammonium as an Anodyne,---------------------- 478
Bromide of Potassium in Summer Complaints,---------------482, 616
Belladonna in Hooping-Cough,-- 497
Berlin, Letter from, ----------- 666
Bronchitis, Chronic, as Connected with Gout, etc., ------------- 122

PAGE.
Calabar Bean, Extract of,------- 764
Contagion and Utilization,------ 32
Clinical Cases of Continued Fever, 46
Clerical Surgeon,--------------- 60
Curvature of Spine, Lateral,----- 77
" " Backward, -- 594
Clinical Reports, Mercy Hospital, 87, 173, 221, 656
Chloroform, Death from, -------- 120
Chloroform in Intermittent Fever, 135
Chloroform, Combination of with Opiates,--------------------- 746
Conspectus of Medical Sciences,-- 177
College Commencement, -----178, 241
Carbolic Acid, Antidote for,----- 184
Charlton, John, Death of, ------- 240
Convulsions, Puerperal,--------- 249
Correspondence,---------------- 310
Catarrhus Vesicæ, -------------- 313
Calcification of Tooth Pulp,----- 315
Creasote in Typhoid Fever,------ 380
Cataract, Cure of, without operation,------------------------ 439
Carbolic Acid; its Surgical and Therapeutic Uses, ------------ 501
Cholera and Yellow Fever in Cuba, 504
Consumption, Contagion of,------ 505
Chicago Medical College, Medical Dept. N. W. University, ------ 634
Canadian Medical Association,--- 634
Clavicle, Excision of, for Oseto Sarcoma,------------------- 653
Cook County Hospital Records,-- 663, 739
Chemical Decomposition Retarded by Pressure,----------------- 655
Cattle Diseases, Report on, ------ 690
Cholera, Prevention of its Spread, 694
Club-Foot, Practical Manual on,- 431
Chemistry, Elementary, Manual of,----------------------501, 690
Compendium, Half-Yearly,------ 313
Convention, Medical,----------- 437
Criminal Abortions, ------------ 757

PAGE.

Clinical Instruction to Mixed Classes, 758

Death, a Case of Sudden, 15
Diabetes, Saccharine, Remark on, 94, 314
Dysentery, Chronic, A New Treatment of, 108
Dental Association, American, 111
Dose and Symptom Book, 121
Dropsy, Renal, with Convulsions, 193
Dunn, A. A., Death of, 240
Discoveries of Sir Charles Bell, 249
Dipsomaniacs, 250
Drugs and Medicines, Report on, 391
Digestion, Function of, 432
Doctors, etc., in Paris, 445
Diarrhœal Diseases of Infants, 551
Disinfectant for Purposes of Dissection, 697

Ectropium Intestinorum, 16
Essentials of the Principles and Practice of Medicine, 176
Errors, 181
Ear, Diseases of, 235
East India Opium, 243
Explanation, 309
Epilepsy, New Investigations Concerning, 330
Editors' Association, 240, 376
Equino-Varus, New Dressing for, 388
Excisions of Head of Femur, Report on, 560
Eye, Diseases of, 560
Eye, Diseases and Injuries of, 756
Etherization in Boston, 565
Earle, F. O., Death of, 636
Ergot, Extract of, 762

Fibrous Tumor of Uterus, removed by W. H. Byford, M.D., 1
Fever Intermittent, 8
" Continued, Strychnine in, 46
" Lectures on, 52
" Typhoid, Evidence of, 154, 570
Fatty Tumor, Case of, 84
Female Sexual Organs, Pathological Anatomy of, 122
Fissure, Anal, Treatment of, 176
Fungus, Theory of Disease Doubtful, 182
Fistula, Vesico-Vaginal, 238
Fracture Apparatus, New, 282
Fossil Horse, Discovery of, 311
Fees in Medical Colleges, 502
Fort Wayne, Letter from, 605

PAGE

Florida and the South, a Guide-Book, 632
Female Students and Physicians, 632
Foreign Gleanings, 695

Gallstone and Cholesterine, Solvent for, 184
Glue, Liquid, 314
Gums, Treatment of, 314
Gynæcological Society, of Boston, 366
Gelsemina, 427

Hæmatemesis, 324
Hæmorrhagic Malarial Fever, 501
Health-Lift, 181
Herpes Zoster, a Case, 157
Hip, Dislocations and Fractures of, 691
Heidelberg, Letter from, 602
Health, Board of, in New York, 561
Hypodermic Injections in Syphilis 455
Hydrate of Chloral, Physiological Action of, 747
Hypodermic Injections in Inertia from Exhaustion, 389
Hypodermic Injection of Caffeine in Poisoning by Morphia, 700
Hemorrhage, Dental, 328
Homœopathy, 309
Homœopathy, Modern, 766
Horse-Shoe Pessary, Removal of from Female Bladder, 729
Hotel Dieu, Mortality in, 439

Ileitis, a Case, 577
Indians, Military Surgery Among, 599
Insanity, Report on, 513
Infancy, Hygiene of, 23
Institution, Royal, 183
Infancy and Childhood, Diseases of, 235
Items from the Gazette de Hospateaux, 693

Jurisprudence, Medical, in Mass. Medical College, 181
Jurisprudence of Medicine, 505

Knoxville, Letter from, 294
Keratitis, 725

Lexicon, Medical, 121
Larnyngoscope, Use of, 122
Lithotomy, 137
Life in Depths of Ocean, 171
Locomotor Ataxia, Phosphorus in, 232
Lithotomy, Spontoneous, 313
Law, Medical, in Minnesota, 378

PAGE.
Lead Poisoning, 438
Lachrymal Affections, 501
Library, National, 563
Lister's Antiseptic Treatment in Surgery, 606

Medical Society, Chicago, 18, 89, 147, 150, 207, 299, 310, 433, 548, 623
Medical Society, Wisconsin State, 36
" " Carroll County, 93, 217, 499
" " Illinois State, 123 178, 239, 310, 375, 395, 691
" " Wayne County, 218
" " Adams County, 310
" " DeWitt County, 339
" " Iowa State, 414
" " Minnesota State, 498
" " Nebraska State, 692
Mortality Reports, 39, 59, 185, 312, 442, 506, 569, 635, 698, 763
Money Receipts, 58, 124, 245, 311
Medical Instruction, Spring and Summer, 123
Microscope, New Mode of Preparing Objects, 250
Medical Vacancies in the Army, 370
Microscopical Society, Ill. State, 378
Medical Institute, Summer, 433
March, Alden, Death of, 436
McNaughton, James, Pres. Albany Medical College, 700
Male Fern in Tænia, 700
Maxilla, Superior, Excision of, 452
Mercy Hospital, 502, 525, 562
Medical Controversies, 502
Medical Colleges, 562, 692
Military Surgery in French Army, 590
Membrana Tympani in Health and Disease, 631
Mineral Waters, Artificial, 682
Muscles, Physical Properties of, 440
Needle, Extraction of, 329
Nature and Time in the Cure of Disease, 237

Nothing New, 57
Nurses, New York Med. College, 58

Obstetrics, Report on, 457
Opium, Its Action on Uterus, 420
Ophthalmic Surgery, Handy-Book of, 757
Opium Habit, 52
Orchitis, Acute, 379
Oxygen Mixture for Inhalation, 284

PAGE.
Ovarian Dropsy, Operation for, 140
Ovary, Microscopic Examination of, 143
Ovariotomy, 183
Organic Life, Forces of, 705

Physicians' Visiting List, 1869, 691
Pathological Specimens, Method of Preserving, 697
Pharmacist, 634
Paris, Letter from, 534
Placenta Previa, Cases, 569
Pyrophosphate of Iron, Preparation of, 430
Pathological Museum of Pennsylvania Hospital, 432
Pancreas, Minute Structure of, 766
Pharmacy, Chicago College of, 413
Pharmaceutical Association, A'm, 433
Paralysis after Chloroform, 438
Pruritus Vulvæ, 445, 762
Prize, Surgical, 314
Purpura and Tetanus, a Case of, 199
Practical Painter, 239
Pennsylvania Hospital Reports, 236
Pensions to Widows of Physicians, 184
Potassa, Permanganate of, 765
Primipara, Aged, 188
Physiology and Hygiene, a Treatise on, 52
Peticolas, Arthur E., 58
Pseudo-Membraneous Croup, Tracheotomy in, 321

Quacks in London, 184
Quinine, Sweet, 504

Rheumatic Fever, Treatment of, 697
Reply of Dr. Breed to Dr. McElroy, 716

Sulphites as Anthelminitics, 699
Sterility, Pathology and Treatment of, 669
Surgery, Science and Art of, 631
Spine, Backward Curvature of, 594
Soda Sulphis in Fever, 8
Surgeon General's Annual Report, 53
Spirograph, 110
Sanitary Statistics of Chicago, 112
Singular Disease, 313
Sun Stroke, Pathology and Treatment of, 227
Sweating, Excessive, 230
Sterility, Microscope in, 238
Sulphates of Soda, Potassa, etc., 285
Spectroscope, Last Wonder of, 314, 439

PAGE.
Syphilis and Local Contagious Disorders, 236
Syphilis, Subcutaneous Injections in, 381
Stevens, Alexander H., Death of, 381
Skin, Structural Lesions of, 432, 628
Skin, Arsenic in Diseases of the, 765
School of Medicine, Kentucky, 434
Smithsonian Institute, Annual Report of, 756
Saccharite of Iron, 441
Small-Pox, Second Attacks of, 249

Troy, Letter from, 664
Tapeworm, Treatment of, 699, 700
Tetanus, Treatment of, Calabar Bean in, 385
Tracheotomy in Croup, 321
Transactions of College Physicians and Surgeons, Philadelphia, 237
Transfusion, Favorable Result of, 766

University of Pennsylvania, History of, 237
Urethra and Urinary Fistula, Pathology and Treatment of Stricture of, 757

PAGE.
Urethroscope, 183
Urinary Organs, Clinical Lectures on Diseaser of, 176
Urine, Retention of, Extraordinary Case, 85
Uterus, Reflex Irritation from the Rectum as a Cause of some Affections of, 371

Vienna, Letter from, 144, 204, 479, 743
Venesection, 238
Vaccination, Protective Power of, 129, 316
Variocele, Treatment of, 449
Vaginismus, 434
Vaginitis, 440

Women, Diseases of, 309
Webster's Dictionery, 316
Water, Impurity of, 505
Water, How to Test the Purity of, 168
Woman, Physical Life of, 689
Women and Children, Hospital for, 562
Warts. 762

Zymotic Diseases, Prevention of, 83

